# Podoplanin Immunopositive Lymphatic Vessels at the Implant Interface in a Rat Model of Osteoporotic Fractures

**DOI:** 10.1371/journal.pone.0077259

**Published:** 2013-10-09

**Authors:** Katrin Susanne Lips, Vivien Kauschke, Sonja Hartmann, Ulrich Thormann, Seemun Ray, Marian Kampschulte, Alexander Langheinrich, Matthias Schumacher, Michael Gelinsky, Sascha Heinemann, Thomas Hanke, Armin R. Kautz, Matthias Schnabelrauch, Reinhard Schnettler, Christian Heiss, Volker Alt, Olaf Kilian

**Affiliations:** 1 Laboratory of Experimental Trauma Surgery, Justus-Liebig University, Gießen, Germany; 2 Department of Trauma Surgery Gießen, University Hospital of Gießen, Marburg, Justus-Liebig University, Gießen, Germany; 3 Department of Radiology, Justus-Liebig University, Gießen, Germany; 4 Department of Diagnostic and Interventional Radiology, BG Trauma Hospital, Frankfurt/Main, Germany; 5 Centre for Translational Bone, Joint, and Soft Tissue Research, Medical Faculty and University Hospital, Technische Universität, Dresden, Germany; 6 Max-Bergmann-Center of Biomaterials and Institute of Material Science, Technische Universität, Dresden, Germany; 7 Biomaterials Department, INNOVENT e.V., Jena, Germany; 8 Department of Orthopedics and Trauma, Zentralklinik, Bad Berka, Germany; West Virginia University School of Medicine, United States of America

## Abstract

Insertion of bone substitution materials accelerates healing of osteoporotic fractures. Biodegradable materials are preferred for application in osteoporotic patients to avoid a second surgery for implant replacement. Degraded implant fragments are often absorbed by macrophages that are removed from the fracture side via passage through veins or lymphatic vessels. We investigated if lymphatic vessels occur in osteoporotic bone defects and whether they are regulated by the use of different materials. To address this issue osteoporosis was induced in rats using the classical method of bilateral ovariectomy and additional calcium and vitamin deficient diet. In addition, wedge-shaped defects of 3, 4, or 5 mm were generated in the distal metaphyseal area of femur via osteotomy. The 4 mm defects were subsequently used for implantation studies where bone substitution materials of calcium phosphate cement, composites of collagen and silica, and iron foams with interconnecting pores were inserted. Different materials were partly additionally functionalized by strontium or bisphosphonate whose positive effects in osteoporosis treatment are well known. The lymphatic vessels were identified by immunohistochemistry using an antibody against podoplanin. Podoplanin immunopositive lymphatic vessels were detected in the granulation tissue filling the fracture gap, surrounding the implant and growing into the iron foam through its interconnected pores. Significant more lymphatic capillaries were counted at the implant interface of composite, strontium and bisphosphonate functionalized iron foam. A significant increase was also observed in the number of lymphatics situated in the pores of strontium coated iron foam. In conclusion, our results indicate the occurrence of lymphatic vessels in osteoporotic bone. Our results show that lymphatic vessels are localized at the implant interface and in the fracture gap where they might be involved in the removal of lymphocytes, macrophages, debris and the implants degradation products. Therefore the lymphatic vessels are involved in implant integration and fracture healing.

## Introduction

The insertion of bone substitution materials has been proven to accelerate healing of osteoporotic fractures. Osteoporosis is a systemic disease with a characteristic decrease of bone strength and therefore an increase in fracture risk [1]. Usually, postmenopausal women and aged men contract this disease. Because of the demographic trend elderly people will increase in the population which is why in future more people will suffer from osteoporosis and osteoporotic fractures [1]. The commercially available bone substitution materials are not etiology-adapted and therefore not appropriate for the application in osteoporotic damaged bone. In the last years it has been worked out that modification with strontium [2], [3], bisphosphonate [4] as well as enhanced resorption capacity and changes from solid material to interconnected porous material [5] improves the materials. High resorption capacity goes along with the disposal of the degraded fragments often inserted in cell organelles of macrophages. Macrophages containing biomaterials in intracellular vesicles are able to differentiate into osteoclasts which are responsible for bone resorption [6]. Macrophages are usually removed via veins and lymphatic vessels [7]. The lymphatic network begins with the lymphatic capillaries in the peripheral tissues that transport lymph to the venous system via passage through several regional and collective lymph nodes. Lymph consists of interstitial fluid, lymphocytes, and macrophages. Lymphatic capillaries differ from vascular capillaries in their larger diameter, irregular outline, thinner walls, incomplete basal membrane, overlapping endothelial cells, and molecular composition of their endothelial cells [7]. In the last years several markers have been identified, that are qualified for identification of lymphatic vessels [8]. The aim of the present study was to identify the lymphatic vessels in a rat model of osteoporotic fractures which were stabilized by newly established bone substitution materials. Therefore, we recently established a fracture defect model in the distal metaphyseal area of the femur of osteoporotic rats [9-11]. We decided to use small animals in this first approach because of the shorter breeding cycles, easier and less expensive animal housing that allowed us to investigate several different bone substitution materials. A follow up study in a large animal model (e.g. ovine model) where only the most suitable biomaterials from this first study will be integrated would be very helpful because of the closer mimic of large animals to the situation in humans. Rat lymphatic vessels were detected by immunohistochemical staining with a monoclonal antibody against podoplanin. Podoplanin is an integral membrane glycoprotein [12] that was first identified in the murine osteoblast cell line MC3T3 [13]. Later it was clarified that podoplanin occurs in mature osteoblasts and newly formed osteocytes [14]. The name podoplanin is explained by its occurrence in podocytes of the kidney [15], [16]. Besides it is also expressed in tumors (e.g. [17], [18] [19], [20],), reticular and epithelial cells of lymphatic tissues [21], alveolar type 1 cells of the lung [22], [23] [24],, fibroblasts and premalignant keratinocytes of skin [19], [25], stromal cells of placenta [26], choroid plexus, ependymal and neuronal cells of brain [27], [28], and fibroblast-like synoviocytes in rheumatoid arthritis [29]. Because of this widespread distribution the protein is also well known under the synonyms OTS-8 [13], E11 antigen [14], gp38 [21], T1α [22], PA2.26 [19], RANDAM-2 [28], RTI40 [23], and Aggrus [17]. Lymphatic vessels were amongst others found in skeletal muscle [30], heart [31], and during pathological conditions of eye [32], skin [19], [25], and bone [7], [33]. Lymphatic vessels were absent in healthy cancellous and cortical bone but commonly exist in the periosteum and connective tissue [34], [7]. Lymphatic vessels only occur in bone after lesions, fractures, or degenerations [7], [33]. It is supposed that the lympahtics are spreading from the periosteum and the surrounding connective tissue into the damaged bone area. Lymphangiogenesis is stimulated by inflammatory cytokines because of its function in transport of immune cells and clearance of antigen [35]. Pro-inflammatory mediators also promote the formation of hematoma as one of the first steps of fracture healing [36]. The hematoma is infiltrated by immune competent cells (e.g. lymphocytes, granulocytes) and macrophages [36]. The duty of these cells is the removal of debris and the release of cytokines and growth factors that promote fracture healing. Neutrophil granulocytes are usually recruited by lymphocytes that afterwards leave the hematoma through lymphatic vessels. Thus lymphatic vessels are usually appearing during the subsequent stages of fracture healing where lymphocytes, neutrophils, and macrophages have to be removed after they fulfilled their duty. However, there might be a relation between lymphangiogenesis and fracture healing. Implantation of bone substitution materials leads to an improved fracture healing. If bio-degradable material is used macrophages are involved in the resorption and removal of degradation products. An increase in macrophages did not result in a higher number of lymphatic vessels [7]. Also the implantation of plain hydroxyl apatite or its modification with platelet factors did not result in significant differences in the lymphangiogenesis [7]. Nevertheless, lymphatic vessels occurred in the granulations tissue after implantation of both materials. On this background the aim of the present study was to investigate if lymphatic vessels occurred in systematically diseased bone as well as in fracture healing and implant insertion in osteoporotic bone. Furthermore, we asked whether the choice of implant material influences lymphangiogenesis. The obtained results might help for further improvement of implant materials.

## Materials and Methods

### Ethics Statement

All animal procedures were carried out in accordance to the Guide for Care and Use of Laboratory Animals of the National Institutes of Health. The study was approved by the local ethic committee (Regierungspräsidium Gießen, Permit Number: V 54 - 19c20-15 (1) GI 20/28, Nr. 92/2009). All efforts were made to minimize suffering.

### Animal model

We recently established a fracture defect model in the distal femoral metaphysis of osteoporotic rats [9-11]. In brief, 14 week old female Spargue-Dawley rats (Charles River, Sulzfeld, Germany) underwent laparotomy with subsequent removal of ovaries under deep anaesthesia with i. p. injections of ketamine (62.5 mg/kg bodyweight, Hostaket^®^, Hoechst) and xylazine (7.5 mg/kg bodyweight, Rompun^®^, Bayer). The animals were kept under a 12 h light-dark cycle with free access to water and chow that were specifically produced for deficiency diet of calcium, vitamin C/D^2^/D^3^, and phosphate (Altromin-C1034, Altromin-Spezialfutter GmbH, Lage, Germany) [10]. Again under deep anesthesia a wedge-shaped methaphyseal fracture of 3, 4, and 5 mm in diameter was performed at the left distal femur 12 weeks of ovariectomy and the 4 mm defects were filled with one of the implants as described in detail earlier [11], [9]. In brief, a skin incision was made, a 7-hole T-shaped miniplate (Leibinger^®^ XS-miniplate, Stryker^®^, Schönkirchen, Germany) was fixed with 1.7 mm screws on the lateral femur, and the defect was created, rinsed with sterile water, filled with the implant, and closed in multilayers and by prolene 4/0 stitches. Always 7 animals got the same implant (groups) and were sacrificed 6 weeks after surgery. Via the abdominal aorta a lead containing radio-opaque polymer (Microfil^®^ MV-122, Flow Tech, Carver, MA, USA) was infused rinsing the vessels with heparinized saline up to blood freeness (10 ml of 0.9 % sodium chloride with 1000 LU heparin) [11]. In the histological staining the polymerized Microfil^®^ could be found in the lumen of blood vessels. 

### Bone substitution materials

As implant materials calcium phosphate bone cement (CPC) [37] that was composed of 58 wt% of α-tricalcium phosphate [Ca_3_(PO_4_)_2_], 24 wt% dicalcium phosphate (CaHPO_4_), 8.5 wt% precipitated hydroxyapatite [Ca_10_(PO_4_)_6_(OH)_2_], and 8.5 wt% calcium carbonate (CaCO_3_) was used plain and with strontium modification (CPC-S). CPC-S was generated by replacement of CaCO_3_ with strontium carbonate (SrCO_3_) in the cement precursor powder (Sr/Ca = 0.123) [38]. Implantation itself was conducted with a paste formulation of the cement that was created by mixing with an aqueous solution of 4 % Na_2_HPO_4_. 

Furthermore, composites consisting of silica and fibrillar bovine collagen were used for implantation as monolithic xerogels (B30, 70 wt% silica, 30 wt% collagen) or as porous scaffold (pB30, xerogel particles B30, size < 250 µm, embedded in a collagen matrix with xerogel/matrix weight ratio of 1.0) [39], [11], [40]. A modification of the scaffolds was prepared by using xerogel particles consisting of 50 wt% silica, 30 wt% fibrillar bovine collagen, and 20 wt% strontium carbonate (pB30S20). Moreover, iron foam with interconnected pores (Fe) was applied. The iron-foam was basically created of carbonyl iron, Fe_3_-powder, and a polyvinyl alcohol binder by powder-metallurgical replication, subsequent sintering at 1150°C, and shaping by wire eroding. The iron foam was coated with a) SrCO_3_ H_3_PO_4_ (Fe-S) under vacuum for 4 hours (h) and rinsed afterwards with ethanol, and b) with zolendronic acid (Fe-BP) that is a member of the bisphosphonate family. Fe-BP was created by precipitation of zolendronic acid, stearate, and iron. Then, the complex was carefully washed, dried, grinded and finally resulted in a coating of 35 µg zolendronic acid on the basic iron foam. These 8 different formulations of bone substitution materials were implanted in the current animal model.

### Immunohistochemistry

Left femora were fixed in 4% phosphate-buffered paraformaldehyde (Merck, Darmstadt, Germany), dehydrated through a series of ethanol gradient, embedded in methylmethacrylate (Technovit 9100, Heraeus Kulzer, Hanau, Germany), and cut into slices with a thickness of 5 µm using a rotations microtome (Leica RM2155, Wetzlar, Germany). Sections were deplasticized with 3-Methoxyethylacetat (MEA, Merck, 3 x 20 min) and rehydrated through acetone and Tris-NaCl buffer with 0.025 % Triton-X-100 (wash buffer, pH 7.4). Endogenous peroxidase was blocked with 3 % H_2_O_2_ in wash buffer for 5 min. Sections were incubated with primary mouse monoclonal anti podoplanin antibody (antibodies-online, Atlanta, GA, USA) and rabbit anti CD31 antibody (Abbiotec, San Diego, CA, USA) (dilution 1:8000 for podoplanin and 1:350 for CD31; dilution buffer was from Dako, Glostrup, Denmark) at 4°C for 16 hours. After rinsing in wash buffer sections were incubated with biotinlated horse-anti-mouse secondary antiserum (dilution: 1:500) and goat anti-rabbit secondary antiserum (dilution: 1:500) in Tris-NaCl with 1 % bovine serum albumin (Sigma, Taufkirchen, Deutschland) and 12.5 % rat serum (PAA, Pasching, Austria) for 30 min. Afterwards sections were incubated for 30 min in the ABC complex/horseradish peroxidase labeled avidin (Vector laboratories, Burlingame, California, USA) with subsequent development of the staining via incubation in Nova Red (Vector laboratories). Counterstaining of nuclei was performed with hematoxylin (1:3 dilution; Shandon Scientific Ltd, Cheshire, UK) and coverslipped with DePex (Serva, Heidelberg, Deutschland). 

### Quantification of lymphatic vessels

One investigator counted the lymphatic vessels manually using an ocular micrometer. Additionally, a double blind study was conducted where a second investigator confirmed the acquired data. The lymphatic vessels were quantified in the whole fracture gap in sections with empty defects or without residues of the implants. An area of 250 µm at the implant interface was analyzed if the implants remained or at least a residual existed. In a separate set of examinations lymphatic vessels penetrating the iron foam were counted. A manual determination of the lymphatic vessels was necessary because the lymphatic vessels were not uniformly distributed. The evaluation was conducted with a 20 x objective at the light microscope (Zeiss, Jena, Germany).

### Real-time RT-PCR

Tissue from the implant interface and the fracture gap was collected in RNAlater immediately after scarification of animals. Total RNA was isolated with the Lipid Tissue mini Kit (Qiagen, Hilden, Germany) according to manufacturer’s protocol. In brief, 100 mg of the sample was homogenized in 1 ml QIAzol lysis reagents with a ultra-turrax (IKA, Staufen, Germany), 200 µl chloroform were added and centrifuged for 15 min at 4°C. The RNA containing upper fraction was collected and washed with 70 % ethanol and Kit buffer RW1. The dried RNA was resuspended with RNase-free water and reverse transcribed with the Quantitect Kit (Qiagen). Contaminations of genomic DNA were removed using 2 µl DNA Wipeout buffer (component of the Kit) for 2 min at 42°C. Afterwards 1 µg RNA was incubated with 1 µl Quantiscript reverse transcriptase, 4 µl buffer and 1 µl primer mix containing random-primers and Oligo(dT)s at 42° for 30 min. The reverse transcriptase was inactivated at 95°C for 3 min. Real-time RT-PCR was carried out with the Light-Cycler Fast Start DNA Master plus SYBR Green Kit (Roche Mannheim, Germany) in a Light Cycler 2.0 (Roche). 2 µl of cDNA, 2 µl Roche reagent (Kit component), 6.8 µl RNase-free water and 0.2 µl forward and reverse primer (Table 1, MWG Biotech, Ebersberg, Germany) were added and incubated for 10 min at 95°C, following by 40 cycles of 5 seconds (s) heating at 95°C, annealing for 5 s at 58°C and elongation for 5 s at 72°C. The PCR product was controlled by melting curve and gel electrophoresis. As negative controls a) reverse transcription was carried out without adding the enzyme and b) water was used instead of template for conducting PCR. Calculation of Cp-values was performed by Light Cycler software. For quantification Cp-values of podoplanin were normalized with the reference gene β-actin. 

**Table 1:** Primers used for RT-PCR.

targets		sequence	length [bp]	annealing [°C]	Accession
β-actin	fwd^1^	TGTTACCAACTGGGACGACA	165	58	NM_007393.3
	rev^2^	GGGGTGTTGAAGGTCTCAAA			
Pdpn^3^	fwd	ACCCCAATAGAGATAACGCAGG	75	58	NM_019358.1
	rev	CCAGGGTCACTACAGCCAAG			

1 forward ^2^,reverse ^3^,Pdpn = podoplanin

### Statistical analysis

Statistical analysis was performed using the SPSS software (version 21.0; SPSS Institute Inc, Cicago, USA), non-parametric Kruskal-Wallis and Mann-Whitney tests. Confidence level of p ≤ 0.05 was considered to be significant.

## Results

### Osteoporotic fracture model

**Figure 1:**
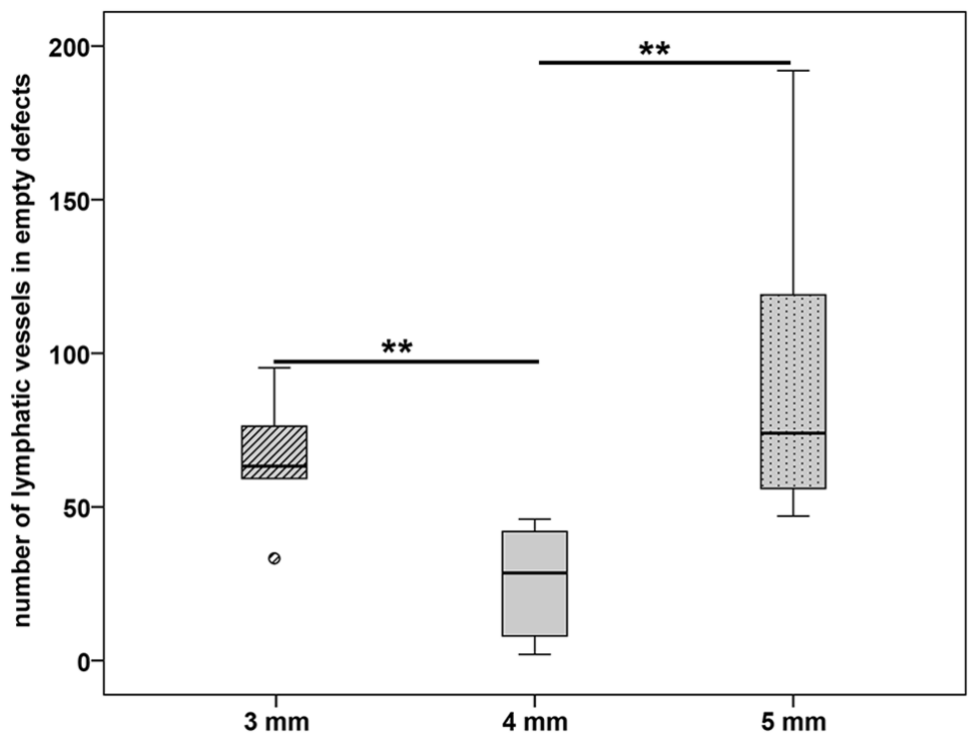
Number of lymphatic vessels in empty defects. The 4 mm sized empty defect contained a significant lower number of lymphatic vessels in comparison to the 3 mm and 5 mm defects. The results are present as box plot where the median is indicated by a solid line within the box, the 25^th^ and 75^th^ percentile as bottom and top of the box, the 0^th^ and 100^th^ percentile as lower and upper whiskers, respectively. Small circles illustrate data beyond 3 x standard deviation (SD). ** = p ≤ 0.01.

**Figure 2:**
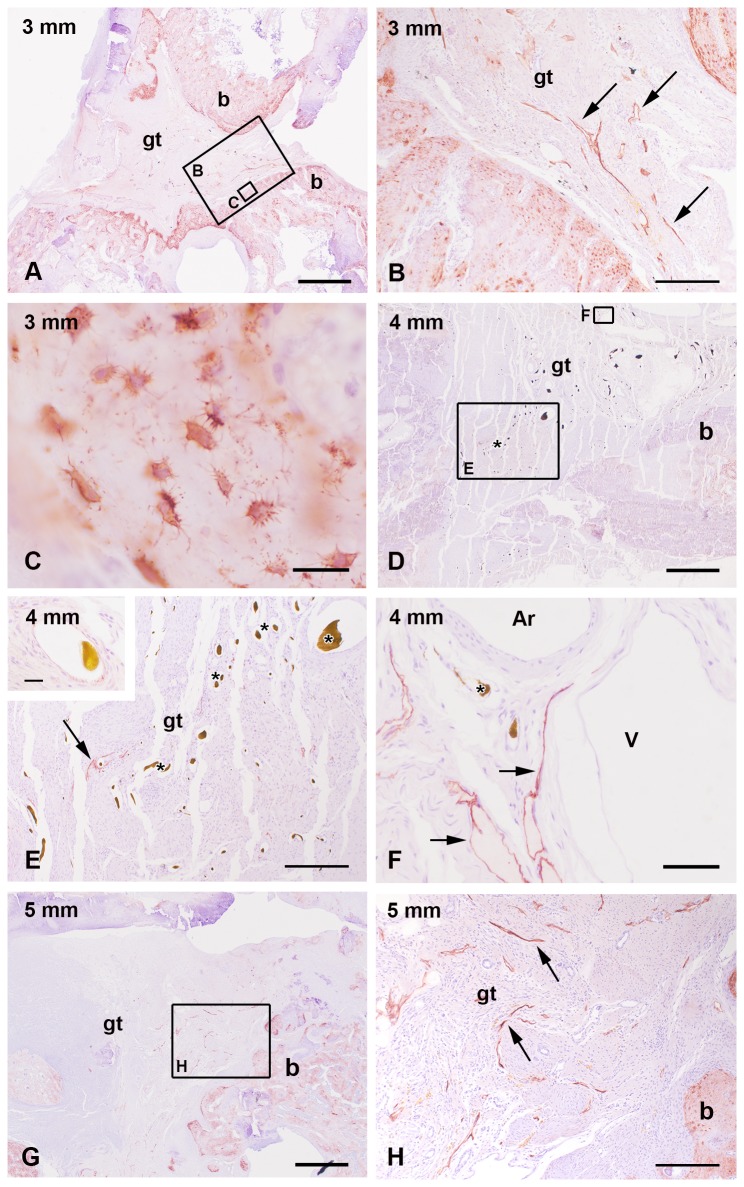
Podoplanin immunohistochemical staining in the rat osteoporotic fracture model. The 3 mm (A) defect was narrowed by new built bone (b) but the remaining granulation tissue (gt) contains a high number of lymphatic vessels (arrow in B). Podoplanin positive osteocytes are localized in the new built bone (C). The 4 mm defect (D) is a typical critical size defect showing no bridging of the fracture gap that was filled with granulation tissue (gt) with only a few lymphatics (arrow in E-F) but a lot of blood vessels (stars). The vasculature was identified by Microfil^®^ perfusion (stars in D-F), immunohistochemistry for CD31 (inset in E), anatomical characteristics (F), and their absence of podoplanin immunreaction (F). The fracture gap of the 5 mm defect (C) was filled with granulation tissue (gt) that contained a high amount of lymphatic vessels (arrow) but only limited blood vessels. Higher magnification of lymphatics showed the irregular shape, the discontinuous endothelium, and thin walls in comparison to the thick muscle layer in the walls of arteries (Ar) and continuous endothelium and wider lumen of veins (V in F). The nuclei were counterstained with hematoxylin. Bar: 1 mm in A, D, G, 200 µm in B, E, H, 20 µm in C, F and inset in E.

**Figure 3:**
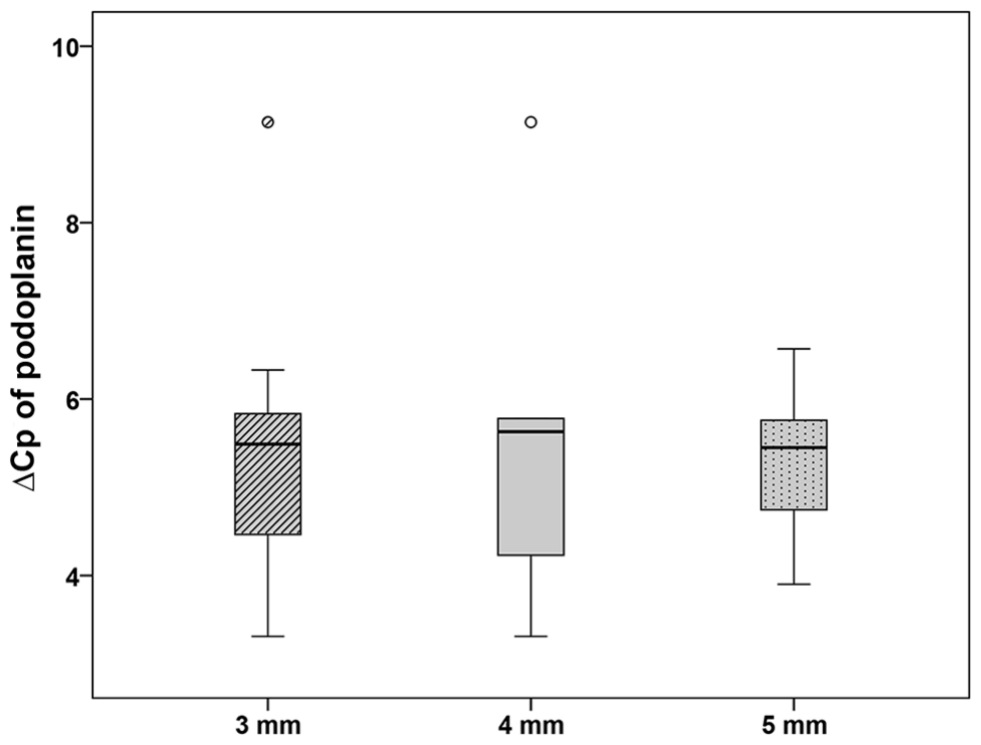
Expression of podoplanin in empty defects. No regulation of the podoplanin expression was detected for the empty defects by means of real-time RT-PCR. The results are presented as box plot with the median, the 0^th^, 25^th^, 75^th^, and 100^th^ percentile. Small circles illustrate data beyond 3 x standard deviation (SD).

Fractures without implanted bone substitution material showed a filling of the fractured gap with granulation tissue that contained several lymphatic vessels. Since we investigated different fracture sizes (3, 4, and 5 mm), we estimated the number of podoplanin immunopositive vessels in the fracture gap. The mean number of lymphatic vessels was significant lower in the subgroup with 4 mm defect size (25.83 ± 7.23) in comparison to the 3 mm (64.83 ± 8.36) and 5 mm (97.00 ± 26.66) defects, respectively (Figure 1). The 3 mm defect contained a high amount of lymphatic vessels inside the small remaining granulation tissue between the parts of the almost bridged fracture gap (Figure 2A-B). The 4 mm defect represented a critical size defect with a fracture gap that was filled with granulation tissue containing only a few lymphatic vessels (Figure 2D-E) but a lot of blood vessels. The 5 mm defect showed a delayed fracture healing. The fracture gap was filled with a high amount of granulation tissue containing a high number of lymphatic vessels but only limited blood vessels (Figure 2G-H). Blood vessels were identified by a) filling with Microfil^®^ (Figure 2D-E), immunohistochemical staining of vascular endothelial cells using an antibody against CD31 (inset in Figure 2E), the absence of podoplanin immunolabelling, and anatomical characteristics (Figure 2F). Contrary to lymphatic vessels with their irregular shape, thin and discontinuous walls arteries in the fracture gap were identified by their regular shape and thicker walls because of the muscle cells in the media (Figure 2F). Veins displayed a broader lumen and a continuous endothelium in contrast to lymphatic vessels in the fracture gap (Figure 2F). The podoplanin immunostaining was also detected in osteocytes of the new build bone (Figure 2C). On mRNA level no regulation was detected between the groups with different fracture size (3 mm, 4 mm and 5 mm, Figure 3)

### CPC based implants

**Figure 4:**
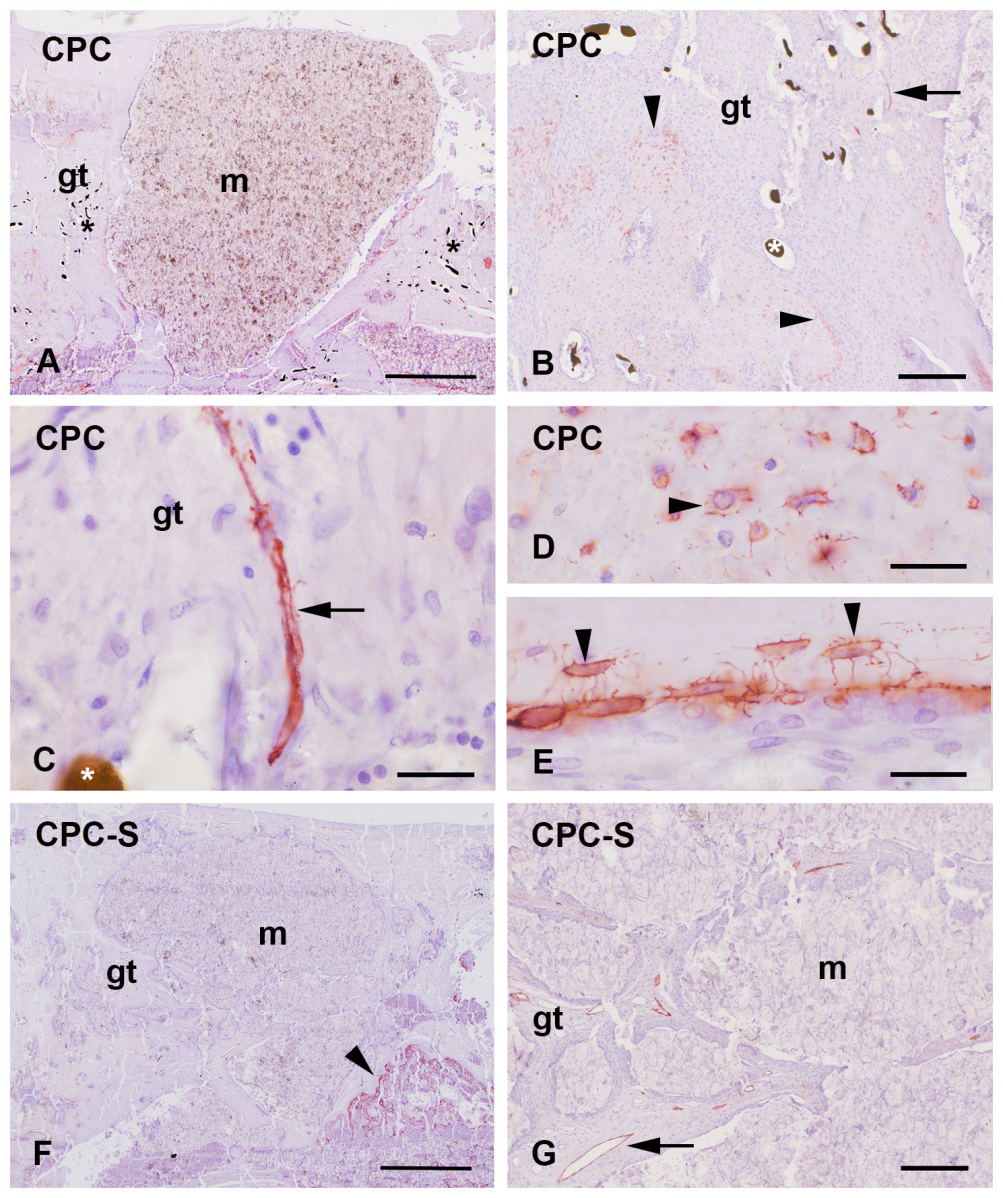
CPC based implants. Podoplanin immunopositive lymphatic vessels (arrow) were localized in the granulation tissue (gt) at the interface of the CPC implant (A-E) as well as at the strontium functionalized CPC (CPC-S in E-F) where an improved fragmentation was found (F). Lympatics were often associated with blood vessels that were identified by Microfil^®^ perfusion (star in C). Podoplanin also stained osteocytes (arrowhead in B-F) and mature osteoblasts (O). Nuclei were counterstained with hematoxylin. m = bone substitution material. Bars: 1 mm in A, F, 200 µm in B, G, and 20 µm in C-E.

**Figure 5:**
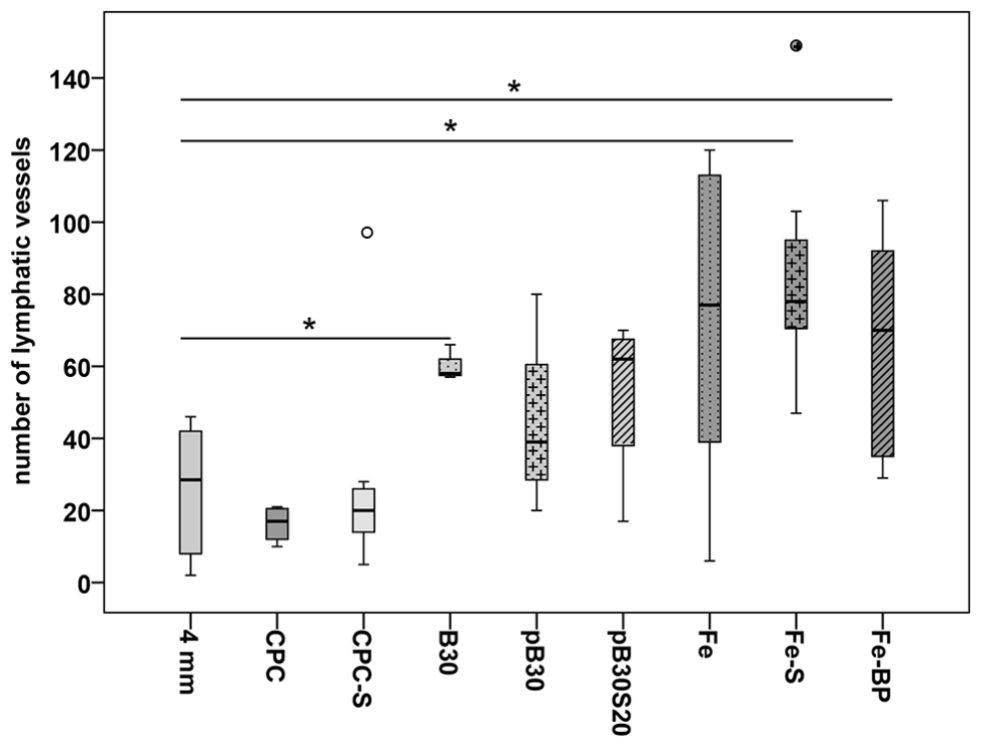
Number of lymphatic vessels at the interface of the implant. A significant up-regulation was found for the silica-collagen xerogel (B30), the strontium (Fe-S) and bisphosphonate (Fe-BP) functionalized iron foam in comparison to the negative control without implant. Data were presented as box plots where a solid line within the box indicated the median. Small circles show data beyond 3 x standard deviation (SD). CPC = calcium phosphate cement, CPC-S = strontium modified CPC, pB30 = composite scaffold, pB30S20 = strontium-modified composite scaffold, B30 = xerogel consisting of 70 wt% silica, 30 wt% collagen, Fe = iron foam. * = p ≤ 0.05.

**Figure 6:**
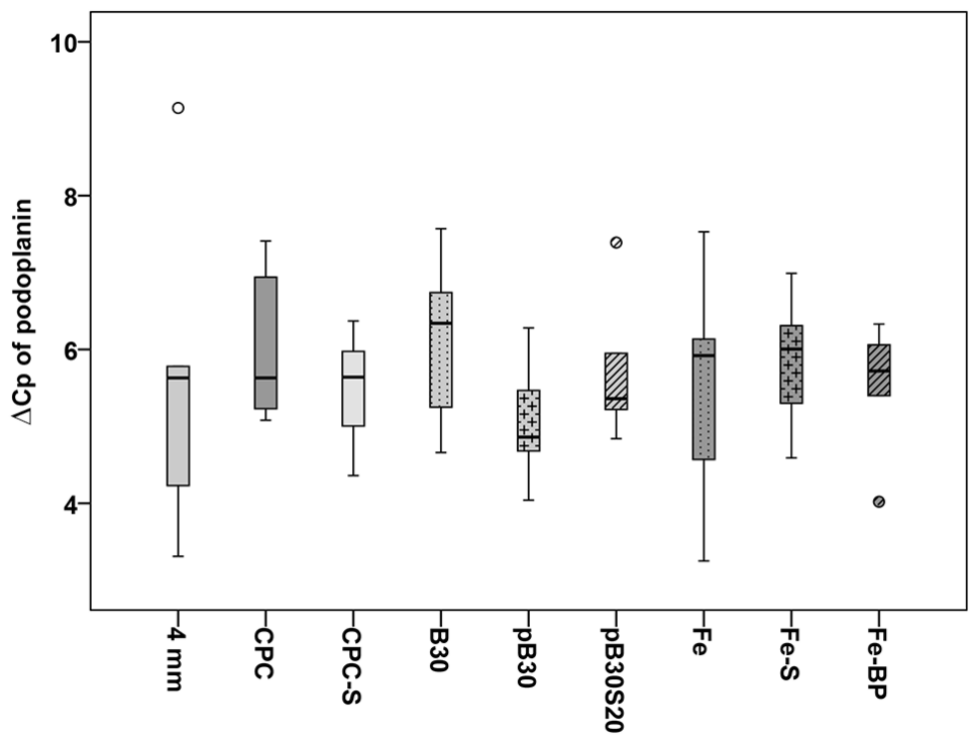
Expression of podoplanin at the implant interface. Using real-time RT-PCR, no regulation of podoplanin at the mRNA level could be detected in the interface area of the implant. The results are presented as box plot with the median, the 0^th^, 25^th^, 75^th^, and 100^th^ percentile. Small circles illustrate data beyond 3 x standard deviation (SD). CPC = calcium phosphate cement, CPC-S = strontium modified CPC, pB30 = composite scaffold, pB30S20 = strontium-modified composite scaffold, B30 = xerogel consisting of 70 wt% silica, 30 wt% collagen, Fe = iron foam.

Two different formulations of the CPC based implants were analyzed: a) plain CPC (CPC, Figure 4A-E) and b) strontium functionalized CPC (CPC-S, Figure 4F-G). Six weeks after surgery the implants were surrounded by granulation tissue that contained blood as well as lymphatic vessels (Figure 4A-C). These two kinds of vessels were often found in association (Figure 4C). The vasculature was not equally distributed around the implant. Some of the CPC-S implants were already fragmented (Figure 4F-G). Then, granulations tissue was found between the implant that contained several lymphatic vessels (Figure 4G). The podoplanin immunoreactivity was also found in mature osteoblasts and osteocytes that were situated in adjacent lacunae surrounded by the calcified bone matrix (Figure 4B, D-F). The labeling was found in accordance to the description of in the plasma membrane as well as the pseudopodia of osteocytes that were situated in the bone canaliculi (Figure 4D-E). The staining intensity of lymphatic vessels and osteocytes was similar (Figure 4B). No significant differences were determined for the number of lymphatic vessels (Figure 5) as well as for the podoplanin expression by comparing CPC, CPC-S and the 4 mm empty defect that served as control (Figure 6).

### Composites of silica and collagen

**Figure 7:**
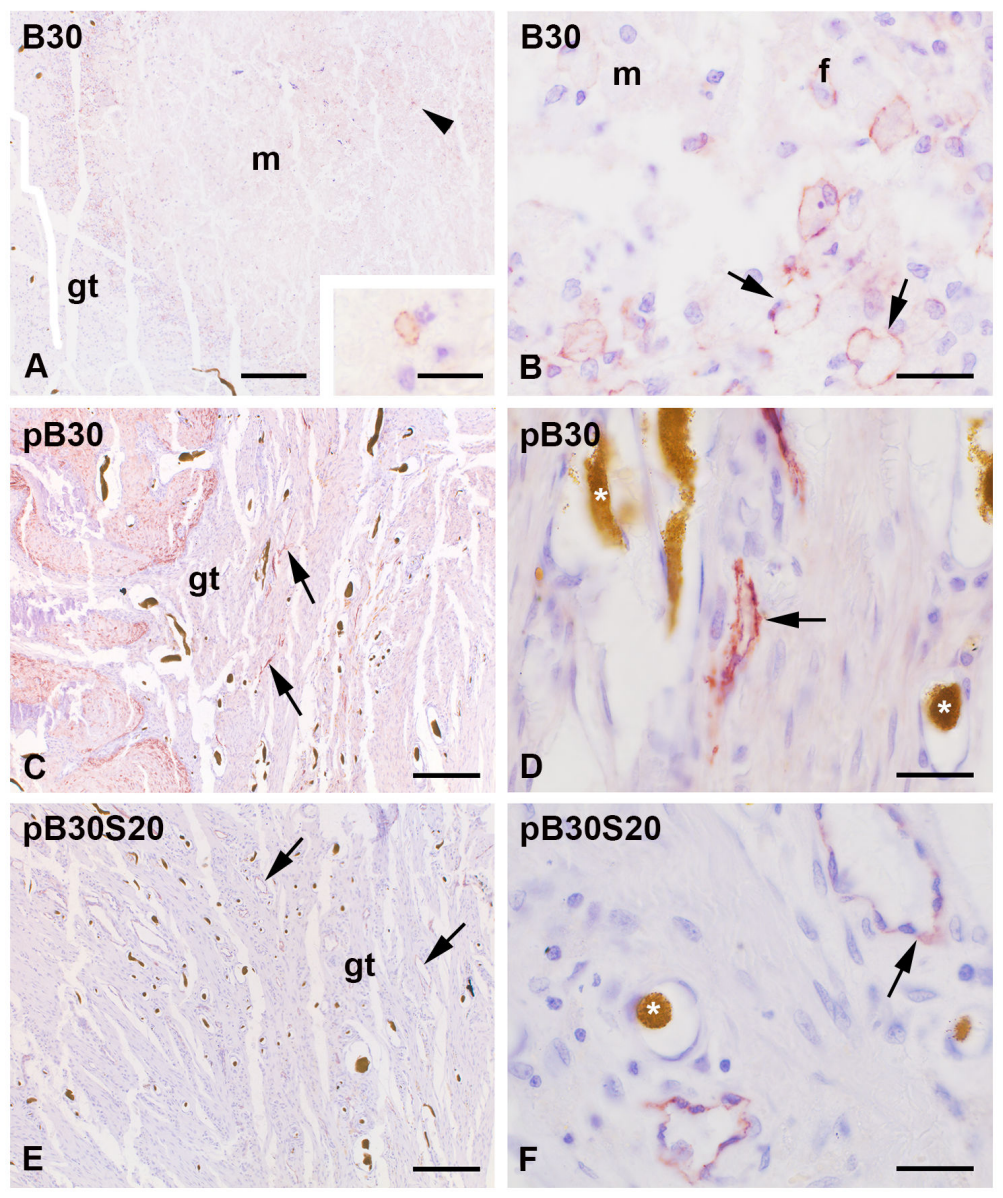
Podoplanin immunoreactivity at composites assembled by silica, collagen (pB30, B30), and additional strontium (pB30S20). The implanted xerogel (B30, A-B) was surrounded by podoplanin immunopositive structures that were related to developing lymphatic capillaries (arrow in B) and additional myofibroblasts (f in B). Some immunoreactivity was also found in the implant (arrowhead and in higher magnification in the inset in A). No residues of the porous composite scaffolds (pB30, pB30S20) were detected (C-F). Podoplanin labeled lymphatic vessels (arrow) were found in the granulation tissue (gt) of the fracture gap (C, E). The lymphatics (arrow) were often localized close to blood vessels (stars in B, D) that were identified by their filling with Microfil® (star). Nuclei were counterstained with hematoxylin. m = bone substitution material. Bar represents 200 µm in A, C, E and 20 µm in inset of A, B, D, and F.

Two types of the composite were used: monolithic xerogels (B30, Figure 7A-B) and porous scaffolds (pB30, pB30S20, Figure 7C-F), both with the dimension of a 4 mm wedge-shape fitting exactly the fracture gap. After 6 weeks of implantation the residue of the partially resorbed xerogel (B30) was surrounded by granulation tissue that contained a high amount of cells (Figure 7A). Podoplanin immunopositive structures were found at the direct interface of the implant and, in addition, penetrating the implant (Figure 7B), where the podoplanin positive structures did not form fully developed lymphatic vessels. It appeared like broken lines, a staining of plasma membrane of big cells, and labeled lymphatic endothelial cells during the process of lymphangiogenesis (Figure 7B). 

In contrast no residues of the porous B30 bone substitution material could be found in the fracture gap (Figure 7C). Instead, the gap was filled with granulation tissue containing several fully developed podoplanin immunopositiv lymphatic vessels (Figure 7D). The same situation was observed for the strontium modified scaffolds (Figure 7E-F).

Comparing the number of lymphatic vessels we could neither detect significant changes between the different composite types nor between the porous scaffolds (pB30, pB30S20) and the empty defect (Figure 5). The xerogel (B30) exhibited a significant (p = 0.024) increase in the amount of lymphatic vessels at its interface in comparison to the control group (4 mm empty defect) (Figure 5). No regulation of podoplanin was found comparing xerogels, porous scaffolds, and the 4 mm empty defect as appropriate control (Figure 6).

### Iron foam implants

**Figure 8:**
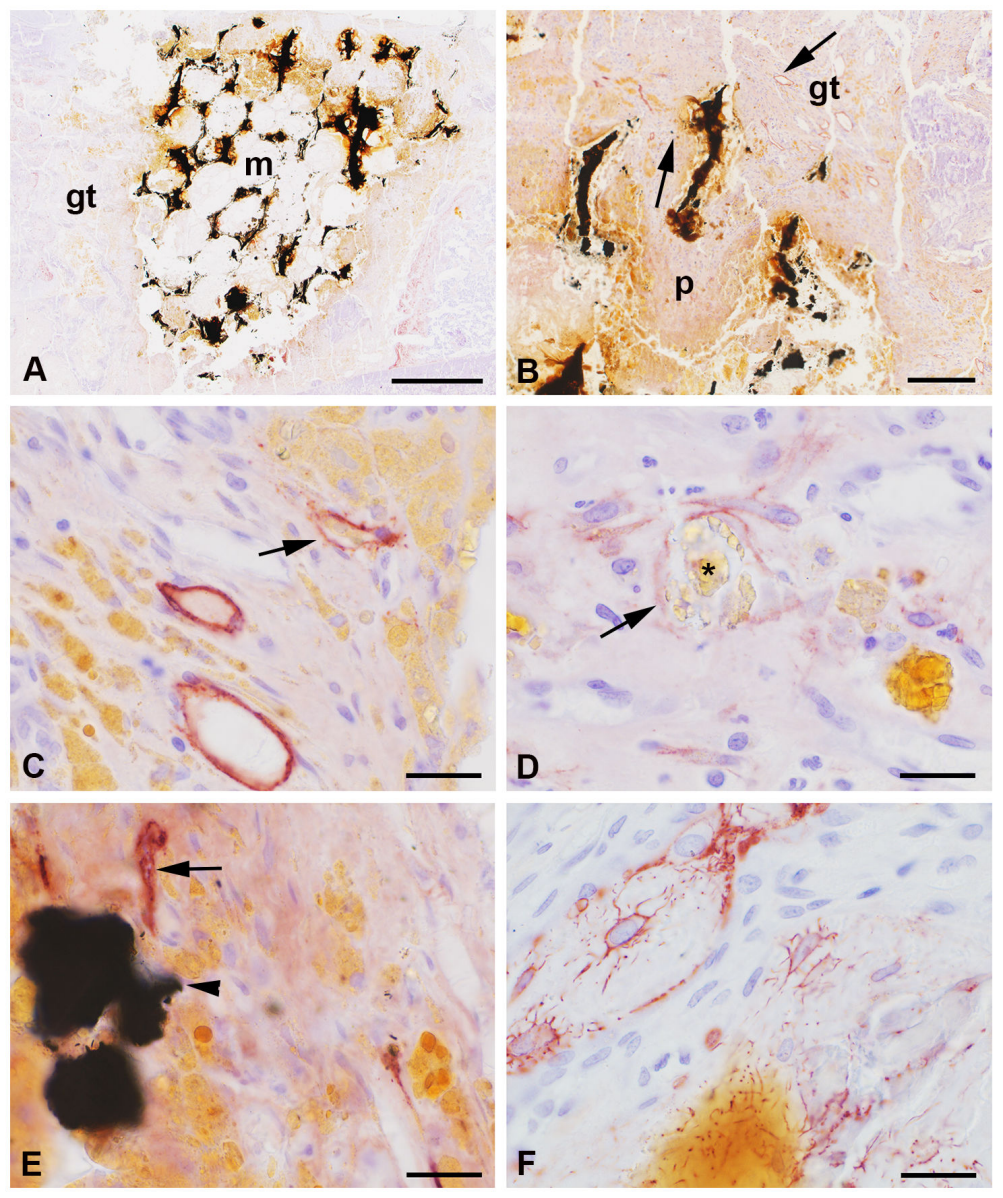
Podoplanin staining at the plain iron foam (Fe). The interface of the wedge-shaped implant (m) was covered with granulation tissue (gt) that infiltrated the interconnected pores (p) near the interface (A-B). Podoplanin immunopositive lymphatic vessels (arrow) were found in the granulation tissue often close to the yellow cells with the resorbed iron (C). Granula of resorbed iron (star) were found in lymphatic capillaries (D). Lymphatics were also situated next to iron fragments (arrowhead, E). Islets with podoplanin stained osteocytes were detected in the granulation tissue (F). Nuclei were counterstained with hematoxylin. Bar represents 1 mm in A, 200 µm in B, 20 µm in C-D.

**Figure 9:**
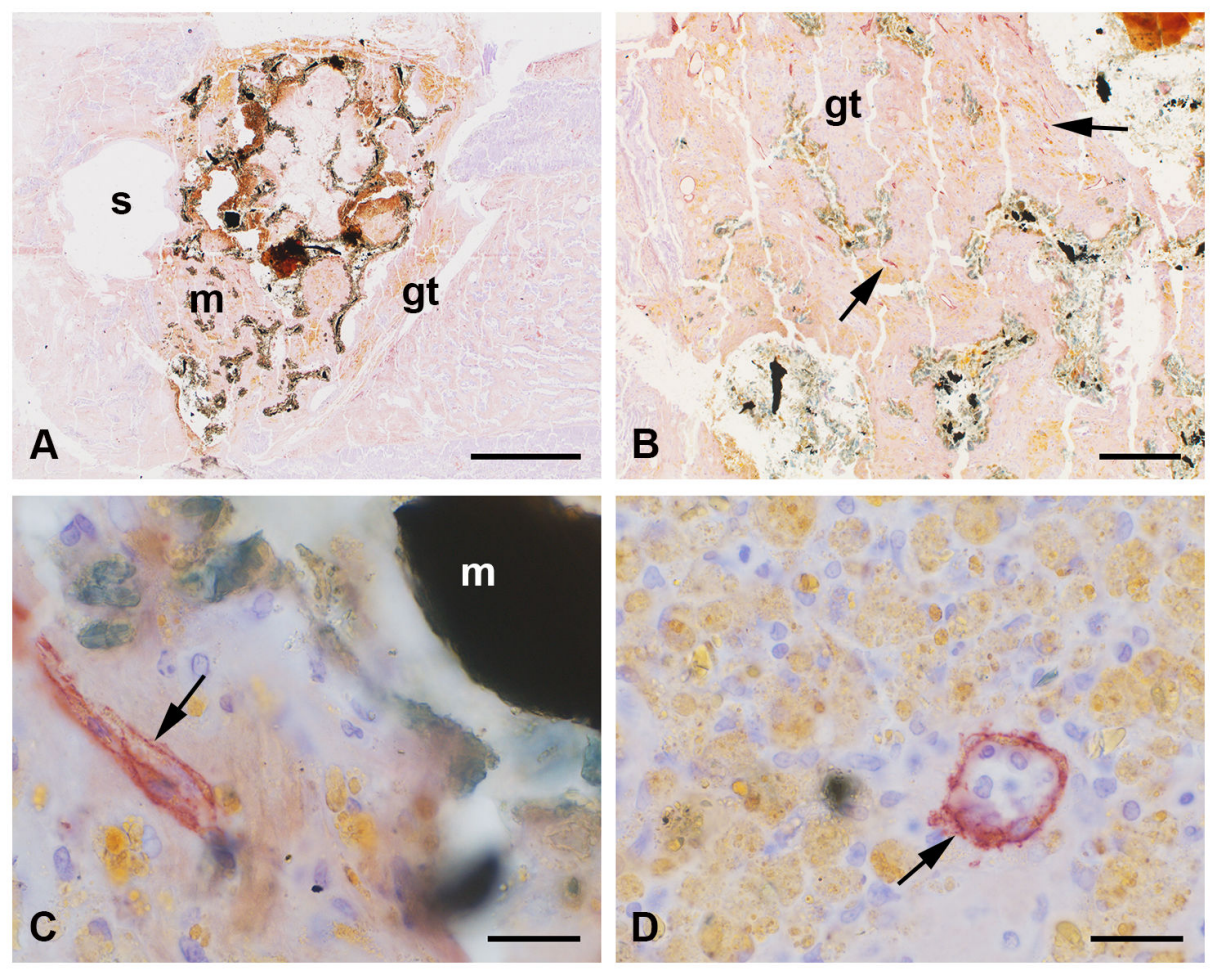
Podoplanin immunohistochemistry at the implant of iron foam with a strontium coating (Fe-S). Podoplanin labeled lymphatic vessels (arrow) were found in the granulation tissue (gt) at the implant (m) interface and in the interconnected pores (A-B). Higher magnification showed the adjacency of the bone substitution material and the lymphatics (C). A high amount of cells with yellow granular cytoplasm were localized in the granulations tissue (D). These cells are supposed to resorb the degraded implant. They are often found close to the lymphatic vessels that sometimes seem to contain lymphocytes (D). Nuclei were counterstained with hematoxylin. Bar represents 1 mm in A, 200 µm in B, 20 µm in C-D.

**Figure 10:**
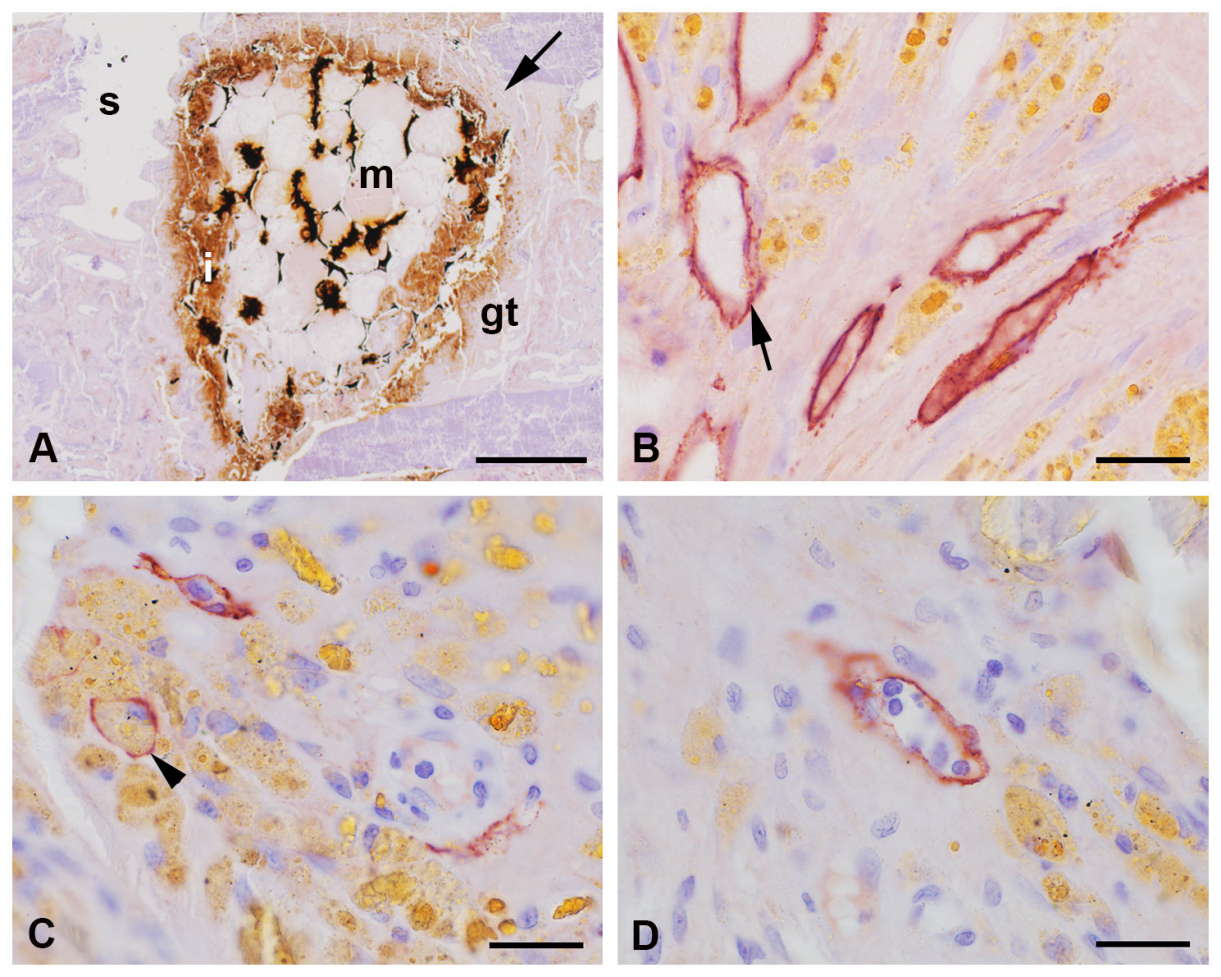
Iron foam coated with bisphosphonate (Fe-BP). Podoplanin immunolabelling was observed in the lymphatic endothelial cells (arrow) that were localized in the granulation tissue (gt) at the implant interface (A-B). Cells with yellow granular cytoplasma that might have phagocytozed the degradation products of the implant were also found at the interface (i). These cells were often situated in direct contact with the lymphatic vessels (B). Few of them were found even in the lumen of the lymphatics (arrowhead in C). Some of the lymphatic capillaries contained lymphocytes as determined by shape and size of their nuclei (D). s = hole of the screw. Bar represents 1 mm in A, 20 µm in B-D.

Three different formulations of the iron foam were used as implant in the different rat groups: a) plain iron foam (Fe, Figure 8), b) strontium functionalized iron foam (Fe-S, Figure 9), and c) iron foam with coating of the bisphosphonate zolendronic acid (Fe-BP, Figure 10). The different modifications of iron foam were surrounded by granulation tissue after implantation (Figure 8A, 9A, 10A). The granulation tissue contained a high amount of fully developed lymphatic vessels (Figure 8B, 9B). No significant differences were found in the number of lymphatic vessels in the granulation tissue (Figure 5). But a significant increase in lymphatics was determined for the modified iron foams (Fe-S, Fe-BP) in comparison to the empty defect (4 mm) that served as control (p = 0.001 for Fe-S and p = 0.026 for Fe-BP) (Figure 5). On mRNA level no significant changes could be measured for podoplanin (Figure 6)

Several cells with yellow granular cytoplasm appeared in the granulation tissue (Figure 8C, 9C-D, 10B-D). This yellow staining could result from resorbed iron. However, these cells are often localized in direct contact to the podoplanin immunopositiv vessels (Figure 10B). Some of the yellow cells were also observed in the lumen of lymphatic capillaries (Figure 10C). In other lymphatic vessels we identified nuclei of lymphocytes (Figure 10D). This was mainly observed at the interface of Fe-BP.

**Figure 11:**
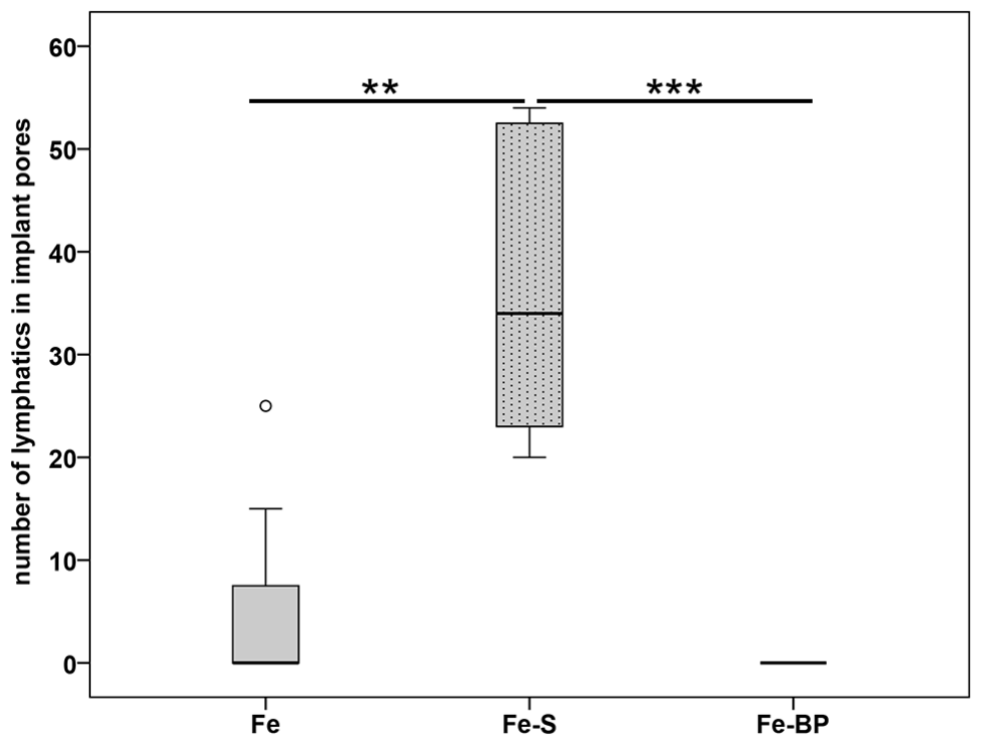
Number of lymphatic vessels localized in the interconnected pores of iron-foams. A significant increase was calculated for the correlation of the counted number of lymphatic vessels localized in the Fe-S implant in comparison to the Fe (p = 0.004) as well as the Fe-BP implants (p = 0.001). Data are presented as box plots with the median indicated by solid line within the box. Small circles illustrate data beyond 3 x standard deviation (SD). ** = p ≤ 0.01, *** = p ≤ 0.001.

Moreover lymphatic vessels were also localized in the connecting pores of Fe and Fe-S but not in the Fe-BP group. These changes in the amount of lymphatic vessels were significant (Figure 11, p = 0.004 and p = 0.001 for the comparison of Fe to Fe-S and of Fe-S to Fe-BP, respectively). The pores of Fe-S were filled with granulation tissue whereas the pores of Fe-BP were not integrated by connective tissue. The interconnected pores of Fe implants were only filled with granulations tissue near the interface. Pores situated in the center of the implant were not filled with granulation tissue after six week follow-up time and therefore no lymphatic vessels occurred there.

## Discussion

The present study demonstrates for the first time the presence of lymphatic vessels in an osteoporosis animal model. Osteoporosis was induced by bilateral ovariectomy and additional feeding with calcium, Vitamin C/D^2^/D^3^ and phosphate restricted diet in current rat model [10]. This treatment is commonly used to induce osteoporosis in rats that are naturally not affected by this disease [41]. Osteoporosis is described as a systemic disease that causes an imbalance of the osteoblast and osteoclast population in which the bone degrading osteoclasts are enhanced [1]. Thereby the bone mineral density is reduced that accounts for the increase in fracture incidence [1]. Recent studies determined that the animals in the applied rat model suffer from a disease with reduced bone mineral density as measured by DXA scan, an enhanced fracture risk as shown by biomechanical analysis, and an imbalance in bone synthesis and depletion [10], [42]. Therefore the rat model can be utilized for studying aspects that are connected to the typical human disease osteoporosis. However, not only the fracture frequency is increased in this illness, but also the fractures are different from such of bone-healthy patients. Characteristically osteoporotic fractures are localized in the spongy bone of metaphyseal trabecular regions [43], [44] like vertebrae, distal radius, proximal femur and humerus [45], [46], [47]. Thus in the present study a wedge-shaped defect in the distal metaphyseal area of the femur was performed [9,11]. We did not dispose the proximal femur because of its bad accessibility. Utilizing the distal metaphyseal area of the femur we assured that the used animal model effectively resets the clinical situation of an osteoporotic fracture as mainly known from aged postmenopausal female patients [11], [9]. Even though an animal model can of course not fully reflect the conditions of humans [41] the current model can be used for analysis of some basic purposes as the occurrence of lymphatics.

The focus of our investigation was the analysis of lymphatic vessels that were identified by immunohistochemistry. Using a monoclonal antibody against podoplanin the discrimination between lymphatic and blood vessels in histological sections is possible and well reproducible [8]. Podoplanin represents a type-1 transmembrane sialomucin-like glycoprotein that is widely spread. In podocytes of kidney the protein is involved in obtaining cell morphology and maintenance in glomerular permeability [16]. Up to now, the function of podoplanin in lymphatic endothelial cells is not fully understood. Nevertheless, similar as in the podocytes the protein might be involved in maintaining the cellular shape and regulate the permeability of lymphatic vessels [15], [16]. 

Lymphatic vessels were not found in healthy adult bone tissue [7], [33] [34], but in the outer periosteum and the surrounding connective tissue [33]. They appear in pathological situations like vertebral destructions, lesions, infections, and degenerations [33]. During early stages of defect healing a hematoma is build up that is infiltrated by macrophages and immune competent cells (e.g. lymphocytes, granulocytes), amongst others [36]. Thus, it was not surprising to find lymphocytes in the lumen of lymphatic capillaries of some animal groups in our implant study. An obvious enhancement of lymphocytes inside lymphatic vessels was determined in groups of plain iron foam (Fe) and Fe-BP. We suppose that these two groups are set in between the hematoma and granulation stage of bone healing. The implant interface already shows fully developed granulation tissue whereas the implant pores were not filled (Fe-BP) or only partially filled (Fe) with granulation tissue. This is contrary to Fe-S implants where the pores were completely filled with granulations tissue. Hence we conclude that strontium ameliorated osseous integration of iron foam and therefore fracture healing. 

Besides, lymphatic vessels are supposed to remove macrophages and their phagocytozed material from the implant interface. Since macrophages with resorbed wear particles from prosthesis were found in lymph nodes it has been assumed that the implant interface is cleared via lymphatic circulation [48], [49], [50]. The removal of macrophages that are containing particles of resorbed material seems to be important because it has been described that this cells are able to differentiate into osteoclasts and secrete prostaglandins, cytokines, and other factors that promote bone resorption [51], [52]. However, in our earlier study we could not determine an up-regulation in the number of lymphatic vessels when the amount of resorbing macrophages increased because of high fragmentation of the implant [7]. Similar like in our previous study the quantification of lymph capillaries was conducted by double blind counting. However, this time two investigators counted the lymphatic vessels of the whole fracture gap, the implant and a region of 250 µm surrounding the implant, and not only in few regions of interest because the vessels were not uniformly distributed. In addition, the occurrence of the lymphatic capillaries could not be compared to implant orientation. No significant accumulation of lymphatics was observed. In general, they were found in the granulation tissue that filled the fracture gap, surrounded and infiltrated the implant. 

As second independent method for quantification we used real-time RT-PCR. However, using this method we could not determine any significant changes (Figure 3 and 6) in podoplanin mRNA (between the different groups). We suspect that the different cell populations which express podoplanin were the reason for these unexpected results. In addition to lymphatic endothelial cells podoplanin is found in differentiated osteoblasts and newly developed osteocytes [14]. Thus, in real-time RT-PCR analysis for RNA isolated out of the whole sample it is not possible to distinguish between lymphatic vessels and the osteoblast lineage. Therefore, we conclude that this second method of quantification was in our case not suitable for the assignation of the number of lymphatic vessels whereas it was well implemented in other studies where no bone was found in the surrounding area [53]. 

In the first part of our study we performed wedge-shaped metaphysal fractures of 3 different sizes. The 3 mm defect was partially bridged by bone containing osteocyts that were localized in adjacent lacunae surrounded by calcified bone matrix. Newly formed osteocytes as well as mature osteoblasts are podoplanin immunopositive [14]. Regardless of the bony bridge some granulation tissue remained and contained a high number of lymphatic vessels inside a small area. The 4 and 5 mm wedge-shaped fractures represent critical size defects [9]. In the fracture gap of the 5 mm defect only new build granulation tissue with a lot of lymphatic capillaries was found. The 4 mm fracture was narrowed by insular regions of newly built bone and the granulation tissue contained a high number of blood vessels. The bony islands were surrounded by granulation tissue that included only few lymphatic capillaries. Also only few lymphatic vessels were found in association with the blood vasculature in the empty defect. Thus we counted for the 4 mm defect a significant smaller number of lymphatic vessels in comparison to the 3 and 5 mm fractures. Our results showed that examination of lymphatic vessels were providing information about bone healing. However, less lymphatic vessels did not reflect best fracture healing as might be assumed because of the absence of lympathic vessels in undamaged bone.

For the second part of our study the 4 mm defects were used to test the occurrence of lymphatic vessels at the interface of bone substitution materials. Applying biomaterials into the fracture gap has been proven to accelerate bone healing [54]. Since up to now none of the commercial available biomaterials is appropriate for the usage in osteoporotic bone we investigated the podoplainin immunolabeling after implantation of several biomaterials. CPC is already well approved for the administration in non-systemically diseased bone tissue [37]. To adapt CPC to the required conditions in osteoporosis it was modified with strontium. Because of the systemic nature of osteoporosis it is not possible that local acting biomaterials themselves influence the illness but modifying them with drugs, growth factors, organic and inorganic active components might promise success. Recently it has been described that strontium administration was successful for the treatment of osteoporosis [2], [3]. Thus one animal subgroup received an implant with a strontium modification of the CPC. At the implant interface of both CPC groups only a few lymphatic vessels were found in close proximity of blood vessels. No significant differences in the number of lymphatic vessels were determined between the two CPC groups themselves, nor in comparison to the control group (4 mm defect). In some animals from the CPC-S subgroup the fragmentation of the implant was upgraded and subsequent granulation tissue including lymphatic vessels penetrated the implant. Thus, in some animals of the CPC-S group we found a higher amount of lymphatic vessels. Nonetheless, no significances were calculated most likely because of the high variance between the individual animals. In another subset of experiments composites consisting of silica and collagen were implanted as monolithic xerogels or as porous scaffolds containing xerogel particles with or without strontium. A recent study demonstrated the composition, structure, surface to have significant impact on the mechanical properties degradation, the homeostasis of calcium, and thereby the local cell and tissue reaction at the interface [55]. Interestingly, we could not discover the porous implant in the histological sections but granulation tissue in the fracture gap that contained lymphatic capillaries. Accordingly, no significant differences in comparison to the control group (4 mm defect) were calculated. However, significant changes were detected for the monolithic xerogels composed of the same ingredients. The number of lymphatics found in this animal subgroup was significant up-regulated in comparison to the control group (4 mm defect). The podoplainin staining in sections of this subgroup had an altered image. The labeled cells were localized directly at the interface. It appeared like an individual labeling of the plasma membrane of a specific cell type. Since it is well known that myofibroblasts are stained by podoplanin [20], [29] and the presence of myofibroblasts at implant interfaces has already been described [56], [57] we hypothesize that at the interface of the xerogel myofibroblasts are accumulated. This interesting theory should further be analyzed studying the osseous integration and degradation of the xerogel in future studies. However, some of the labeling was found in cells invading the implant. An infiltration of the implant was also observed for the Fe-S group. All implants of the Fe group include interconnected pores. Recently, Yoshikawa et al. 2009 [5] described that bone substitution materials with interconnected pores improve bony integration by allowing the immigration of mesenchymal stem cells, granulation tissue, and vasculature into the pores. Interestingly, the pores of the Fe-BP implant were not filled with granulation tissue and therefore no lymphatic vessels were found there. Sections of the plain Fe exhibited a filling of the pores with granulation tissue near the circumference of the implant. Into this area also incorporated lymphatic capillaries were found. The highest number of infiltrating lymphatic vessels was found in the Fe-S group showing the sophisticated implant integration. Besides all Fe implants were surrounded by granulation tissue including lymphatic capillaries where no significant differences were detected.

In conclusion, our results showed that lymphatic vessels also occur in damaged bone of animals where osteoporosis was induced. In addition, we found lymphatic capillaries in the fracture gap and at the interface of several implanted bone substitution materials. After quantification of the lymphatic capillaries we could speculate about the progress of bone healing and implant integration, but most importantly in this study we were able to confirm our hypothesis that also in osteoporotic conditions degraded and by macrophages resorbed biomaterials can be removed from the implant environment by lymphatic vessels. Therefore lymphangiogenesis and migration of lymphatic vessels has been identified as actuating variable for the design of upgraded etiology-adapted bone substitution materials.
